# Alantolactone enhances the sensitivity of melanoma to MAPK pathway inhibitors by targeting inhibition of STAT3 activation and down-regulating stem cell markers

**DOI:** 10.1186/s12935-024-03371-9

**Published:** 2024-05-31

**Authors:** Kun Zhao, Qi Zhao, Xinzhi Dai, Xue Wen, Xing Luo, Yi Duan, Zhihui Yang, Qiong Dai

**Affiliations:** 1 Department of Respiratory and Critical Care Medicine, Xinqiao Hospital, The Army Medical University, Chongqing, 400037 China; 2 Department of Pathology, The Affiliated Hospital of Southwest Medical University, No. 25 Taiping Street, Jiangyang District, Luzhou, Sichuan Province 646000 China; 3 The First Clinical Institute, Zunyi Medical University, Zunyi, Guizhou Province 563003 China; 4 Department of Human Anatomy, School of Basic Medical Sciences, Southwest Medical University, No. 1, Section 1, Xianglin Road, Matan Long District, Luzhou, Sichuan Province 646000 China

**Keywords:** Alantolactone, STAT3, MAPK pathway, BRAF, Melanoma, Drug resistance

## Abstract

Mitogen-activated protein kinase inhibitors (MAPKi) were the first line drugs for advanced melanoma patients with BRAF mutation. Targeted therapies have significant therapeutic effects; however, drug resistance hinders their long-term efficacy. Therefore, the development of new therapeutic strategies against MAPKi resistance is critical. Our previous results showed that MAPKi promote feedback activation of STAT3 signaling in BRAF-mutated cancer cells. Studies have shown that alantolactone inhibited the activation of STAT3 in a variety of tumor cells. Our results confirmed that alantolactone suppressed cell proliferation and promoted apoptosis by inhibiting STAT3 feedback activation induced by MAPKi and downregulating the expression of downstream Oct4 and Sox2. The inhibitory effect of alantolactone combined with a MAPKi on melanoma cells was significantly stronger than that on normal cells. In vivo and in vitro experiments showed that combination treatment was effective against drug-resistant melanomas. Our research indicates a potential novel combination therapy (alantolactone and MAPKi) for patients with BRAF-mutated melanoma.

**Supplementary Information:**

The online version contains supplementary material available at 10.1186/s12935-024-03371-9.

## Introduction

Melanoma is a highly invasive malignancy. The most commonly affected site is skin [1]. At present, the clinical treatment strategy for melanoma is based on extensive surgical resection, combined with chemotherapy, radiotherapy, and biological therapy [2]. Approximately 50% of cutaneous melanomas harbor the BRAF mutations, which trigger excessive activation of MEK/ERK signaling, leading to cell proliferation and immune escape. This is one of the important mechanisms for occurrence, invasion, and metastasis of melanoma [3, 4]. In recent years, US Food and Drug Administration (FDA) has approved 3 groups of MAPK inhibitors (MAPKi) as first line therapies for advanced patients with BRAF mutation. Most patients’ response to MAPKi is rapid and obvious, greatly improving progression-free survival (PFS) and overall survival (OS). However, after 11–14 months, secondary drug resistance emerges in more than 50% of patients [5–7]. In addition, approximately 15-20% of patients with BRAF mutations do not respond to these agents [8, 9]. Ultimately, treatment fails, inevitably limiting the efficacy of therapy. Therefore, there is a critical need to identify the mechanisms involved in resistance to MAPKi and to develop new therapeutic strategies aimed at improving drug sensitivity and conquering primary and acquired resistance.

So far, multifarious molecular mechanisms with regard to resistance of MAPKi have been described, including activation of receptor tyrosine kinases (RTKs), AKT mutations of PI3K/AKT/mTOR pathway and BRAF amplification and splicing variants [10–12]. STAT3 plays a central role in proliferation, metastasis, and drug resistance of tumors [13–15]. STAT3 activation promotes melanoma proliferation, aggressiveness, and metastasis. Our previous study found that MAPKi treatment can activate JAK2/STAT3 signal in BRAF mutant melanoma [16, 17]. Study also showed that activation of HER3/STAT3/SOX2 pathway was the mechanism of resistance against MAPKi in BRAF mutant melanoma cells [15, 18]. Moreover, agents suppressing STAT3 signaling exert anti-melanoma effects [19–21]. These results suggest that inhibition of both STAT3 and MAPK signaling pathways may be a new therapeutic strategy for melanoma.

Alantolactone (ATL), a sesquiterpene lactone derived from Inula helenium, has recently been reported to have therapeutic potential in cancer treatment. Studies have shown that it can inhibit the activation of STAT3 in cancer cells, enhance drug sensitivity, and prevent drug resistance against targeted therapy and chemotherapy [20, 22, 23]. 

To date, no studies have shown the effects of alantolactone on melanoma. Therefore, in current study we hypothesized that alantolactone can inhibit STAT3 activation and increase the sensitivity of melanoma cells to MAPKi treatment. In the present study, we investigated the effects and possible mechanisms of action of alantolactone on BRAF-mutant melanoma with the aim of providing basic research for potential treatment options for melanoma.

## Materials and methods

### Cell lines and reagents

Human melanoma cells A375 and A2058 were purchased from Fenghui Biotechnologies Inc. (Changsha, Hunan, China). HK-2 was purchased from the Beina Chuanglian Biottechnology Reasearch Institute (BNBIO, Beijing, China). A protocol for the establishment and culture of vemurafenib A375R cells was previously reported [16]. A375, A375R and A2058 cells were grown in Dulbecco’s modified Eagle’s medium (High Glucose) (Gibco, Rockford, IL, USA). HK-2 cell was cultured in DMEM/F12 medium (Gibco/life Technologies, Grand Island, NY). All culture media cointained 10% fetal bovine serum (FBS; Gibco, USA) and 1% penicillin/streptomycin (Thermo Fisher Scientific, MA, USA). Vemurafenib was purchased from Shanghai Yuanye Bio-Technology Co. (Shanghai, China). Dabrafenib was purchased from DC Chemicals (Shanghai, China). Cobimetinib and trametinib were purchased from CSNpharm (Chicago, IL, USA). Alantolactone was purchased from Weikeqi Biotechnology Co. (Chengdu, Sichuan, China). All inhibitors were diluted with dimethyl sulfoxide (DMSO) and stored at -80 ℃.

### Viability assays

Cells were seeded in 96-well plates with three replicates per group (A375: 1000 cells/well, A375R: 3000 cells/well, A2058: 4000 cells/well, HK-2: 4000 cells/well). After adherence overnight, cells were treated with different inhibitors or DMSO. After 3 days of processing, cell counting kit-8 (CCK8; Bimake, Houston, TX, USA) was used to detect cell viability according to the manufacturer’s instructions. The absorbance of each well was measured at 450 nm.

### Flow cytometry analysis

Cells were seeded into 6-well plates at 15 × 10^4^ cells/well. Starting from the second day, cells were treated with different inhibitors or DMSO for 24 h and then stained with Annexin V-FITC and propidium iodide (PI) (Dojindo, Kumamoto, Japan) at room temperature in the dark for 15 min, analyzed by the FlowJo software (Ashland, OR, USA).

### Quantitative real-time PCR for relative RNA levels

Total RNA was extracted using the RNeasy Plus Mini Kit (Qiagen, Hilden, Germany) according to the manufacturer’s recommendations. The reverse transcription reactions were performed using the Primescript RT Master Kit (Takara, Dalian, China). Real-time PCR was performed using SYBR Green PCR Master Mix (Bimake, Houston, TX, USA) in the ABI 7500 Fast Real-Time system (Applied Biosystems; Thermo Fisher Scientific, Inc.). Primers are listed in Supplementary Table 1. The 2−∆∆CT method was used to determine the relative expression of target genes after normalization to β-actin.

### Western blotting for protein levels

The detailed steps of protein extraction and western blotting were as previously reported [16, 17]. The following antibodies were used: Anti-ERK1/2 (1:1000, CST, Cat. No. 4695), anti-phospho-ERK1/2 (1:1000, CST, Cat. No. 4370), anti-STAT3, (1:1000, CST, Cat. No. 4904), anti-phospho-STAT3(705) (1:1000, CST, Cat. No. 9145), Anti-Sox2 (1:1000, Boster, Cat. No. BA3292), anti-Oct4 (1:1000, CST, Cat. No. 2750), anti-c-Myc (1:1000, Selleck, Cat. No.A5011), anti-Klf4 ( 1:1000, Selleck, Cat. No. A5391), Anti-Cyclin D1(1:1000, Selleck, Cat. No. A5035), anti-Cleaved PARP(1:1000, Selleck, Cat. No. A5034), α-tubulin (1:2000, Selleck, Cat. No. A5105) and goat anti-rabbit IgG-HRP Antibody (1:4000, ZSGB-BIO, Cat. No.ZB-5301).

### Plasmid transfection

The shSTAT3 Plasmid were obtained from Tsingke (Beijing, China). The plasmid transfection using TSnanofect V1 Transfection Reagent (Tsingke, Beijing, China) was performed as the manufacture’s protocol.

### In vivo xenograft growth studies

Sub-cutaneous xenograft tumors were generated for A375 and A375R cell lines with 2.5 × 10^5^ cells/animal in the right hind legs of 4-week-old male BALB/c nude mice (SPF BIOTECHNOLOGY Co, Beijing, China). Inhibitor treatments were performed by subcutaneous injection for the indicated number of days. Tumor volumes and mice weight were recorded every other days. Animals were excluded if they showed overt toxicity, or lost > 15% body-weight. For molecular analysis of inhibitor effects, six mice per group were treated with vemurafenib (25 mg/kg once daily), Cobimetinib (1 mg/kg once daily) or alantolactone (10 mg/kg once daily) as monotherapy or combination therapy. The longitudinal (L) and transverse (W) diameters of the tumour grafts were measured, and the tumour volume (V) was calculated according to the formula: V= L×W ×W/2. All applicable institutional guidelines for the care and use of animals were followed (SWMU20220037).

### Hematoxylin and eosin staining and immunohistochemistry

Heart, liver and kidney tissues were fixed in phosphate buffer solution containing 4% paraformaldehyde and then embedded in paraffin. Tissue Sect. (4 μm) were stained with Hematoxylin -Eosin staining (Baso, Zhuhai, China), according to the instructions and viewed through a microscope.

Briefly, after deparaffinization and hydration, using specific reagents supplied in the commercial kit, sections were submitted to antigens recovering in the microwave during 5 min. Endogenous peroxide was inactivated for 10 min and unspecific epitopes were blocked with5% Tris-buffered saline (TBS)-BSA buffer during 30 min at 37ºC. Sections were incubated with 1:100 diluted anti-pSTAT3(705) (9145, Cell Signaling Technology, USA); anti-pERK1/2 (4370, Cell Signaling Technology) and anti-c-Myc antibody (TA500002, Zsbio, China), 1:50 diluted anti-Sox2 antibody (TA302025, Zsbio), and anti-Ki67 antibody (TA800648, Zsbio) 1:50 diluted with 2% TBS-BSA. Incubation with the secondary antibody and development with 3,3‐diminobenzidine were done using specific reagents supplied in the kit. After dehydration, tissue sections were counterstained with hematoxylin (Baso, Zhuhai, China) and mounted on slides. Analyses were performed using a high-power objective (10x) on the microscope.

### Statistical analysis

Statistical analyses were performed using SPSS 22.0 software and Graph Pad Prism 8 software. The measurement data were expressed as the mean ± standard error of the mean. The difference between groups was compared using t-tests. The difference among multiple groups was compared using one-way analysis of variance with Tukey’s multiple comparison tests. Each experiment was performed at least 3 times. A value of *P* < 0.05 was considered to indicate statistically significant differences.

## Results

### Alantolactone inhibited proliferation, promoted apoptosis of A375 cells by suppressing STAT3 activation feedback induced by BRAFi

**Fig. 1:**
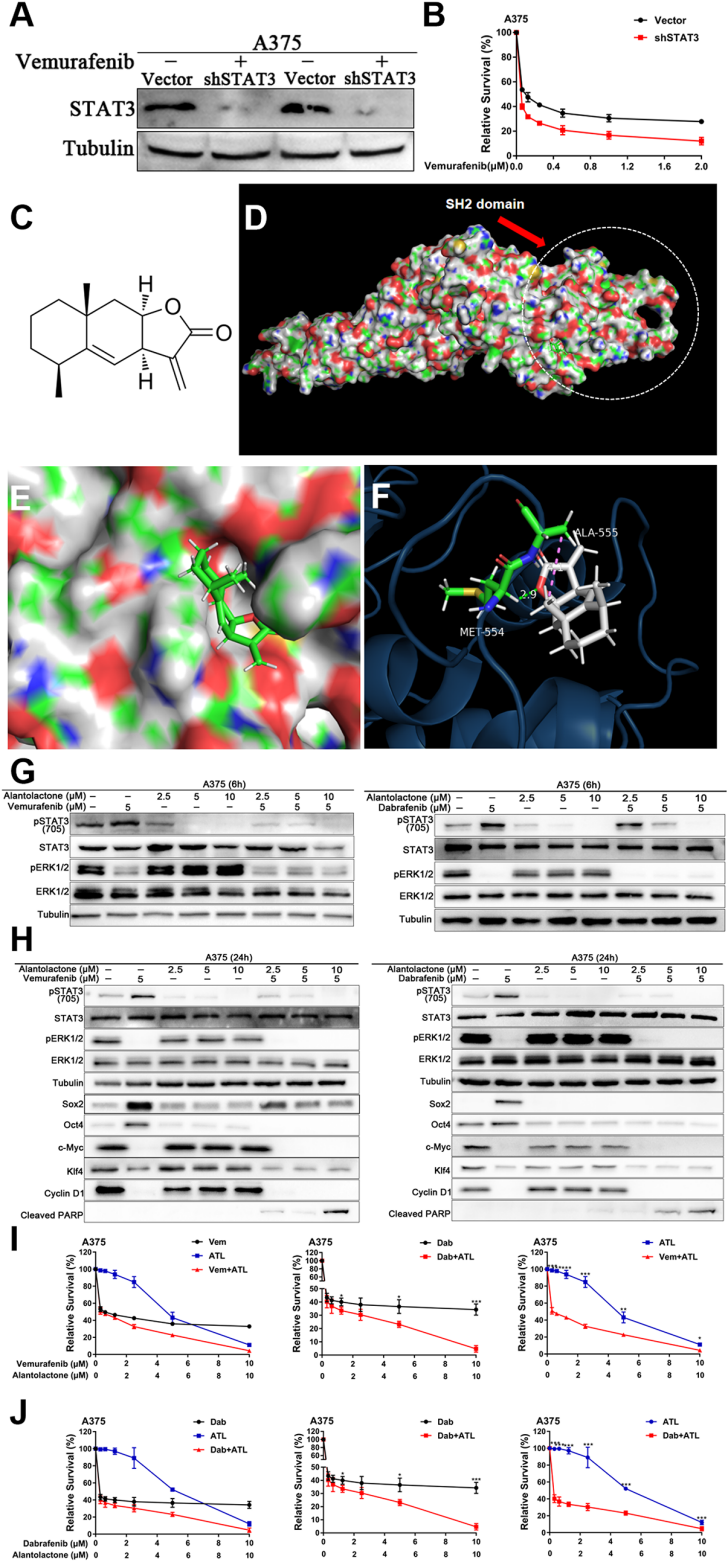
Alantolactone suppressed STAT3 feedback activation induced by BRAFi, downregulating protein expression of Oct4 and Sox2 in A375 cells. (**A**) A375 cells were transfected with shSTAT3 plasmid, and the expression of STAT3 protein was detected 48 h later. (**B**) A375 cells were transfected with shSTAT3 plasmid for 24 h and treated with different concentrations of vemurafenib. After another 72 h, cell viability was detected by CCK8 assays. (**C**) Chemical structure of alantolactone. (**D**) Predicted model of alantolactone binding to STAT3β SH2, as shown by computational modeling. Protein structure information was obtained from Protein Data Bank (PDB) entry 6NJS. (**E**) Binding model of alantolactone to the SH2 domain. The molecular surface of the STAT3β SH2 domain is electrostatically colored with blue and red representing potentially positively- and negatively-charged regions, respectively. (**F**) Predicted interactions between the amino acid residues of the SH2 domain and alantolactone. Oxygen atoms of alantolactone are shown in red. Alantolactone forms carbon hydrogen bond with MET554, and there is a alkyl bond between alantolactone and ALA555. (G and H) A375 cells were treated with alantolactone and BRAFi single or combination for 6 (**G**) or 24 (**H**) hours, phospho-STAT3 (705), STAT3, phospho-ERK1/2, ERK1/2, SOX2, Oct4, c-Myc, Klf4, cyclin D1 and cleaved PARP levels were analyzed by western blotting, and tubulin served as a loading control. (I and J) A375 cells were treated with different concentrations of alantolactone and vemurafenib (**I**) or alantolactone and dabrafenib (**J**) for 3 days. Cell viability was determined by CCK8 assays. The two images on the left in Figures G and H are split from the image on the right. Data are mean ± SD. *p < 0.05; **p < 0.01; ***p < 0.001; Student’s *t*-test

Our previous research found that targeted inhibition of the MAPK pathway promotes compensatory activation of STAT3 signaling in melanoma cells [17]. It is not clear whether inhibiting STAT3 signal increases the sensitivity of melanoma cells to targeted drugs. Our results demonstrated that silencing STAT3 increased the sensitivity of A375 cells to BRAF inhibitors (Fig. 1A and B). The formation of dimers by STAT3 protein is an important step in its activation and regulation of downstream genes. The recognition of specific amino acid residues in the SH2 domain promotes the formation of dimers. Targeting SH2 domain is an important strategy to inhibit STAT3 activation [24, 25]. Virtual molecular docking was used to verify the ability of alantolactone to bind to the SH2 domain. The docking model indicated that alantolactone could form hydrocarbon bonds with amino acid residues in the SH2 domain (Fig. 1C-F). To verify whether alantolactone can prevent the compensatory activation of STAT3 triggered by BRAFi, we treated A375 cells with BRAFi (vemurafenib or dabrafenib) and alantolactone. The phosphorylation level of STAT3 protein was noteworthy decreased compared to cells with BRAFi alone (Fig. 1G and H).

Cell reprogramming is a potential strategy for cancer therapy. Introducing Yamanaka factors (Oct4, Klf4, Sox2 and c-Myc) into cells is a common reprogramming method. We wondered whether melanoma cells would reprogram spontaneously during targeted therapy, so we detected the expression of Yamanaka factors in A375 cells treated with BRAFi. As shown in Fig. 1H, they were altered consistent with pERK1/2 or pSTAT3 (705) protein expression level respectively. The results suggested that Sox2 and Oct4 were the downstream effectors of STAT3, when STAT3 activation was inhibited by alantolactone, Sox2 and Oct4 protein expression level were descended, however, c-Myc and Klf4 were regulated by BRAF/MEK/ERK1/2 pathway.

For further confirmation the actions of alantolactone on A375 cells proliferation and apoptosis. The Cleaved PARP protein of apoptosis marker were detected. In cells of combined alantolactone and BRAFi treatment, the protein expression level of Cleaved PARP was enhanced compared to control cells or cells with single drug. These results indicated that the dual effects of alantolactone and BRAFi promoted A375 cells apoptosis. The A375 cells were treated with different concentrations of alantolactone and BRAFi (Vem or Dab) for 3 days, the cell survival rate was tested by CCK8 assay. The relative survival rate of cells were significantly descending in cells exposured to cooperation of alantolactone and BRAFi, compared with the single BRAFi or alantolactone exposure (Fig. 1I and J). These results verified that the combination of alantolactone and BRAFi has a synergic effect in vitro on inhibiting A375 cells proliferation.

### Dual targeting STAT3 and BRAF/MEK/ERK1/2 with alantolactone and MAPKi had a combined kill effects in vitro on A375 cells

**Fig. 2:**
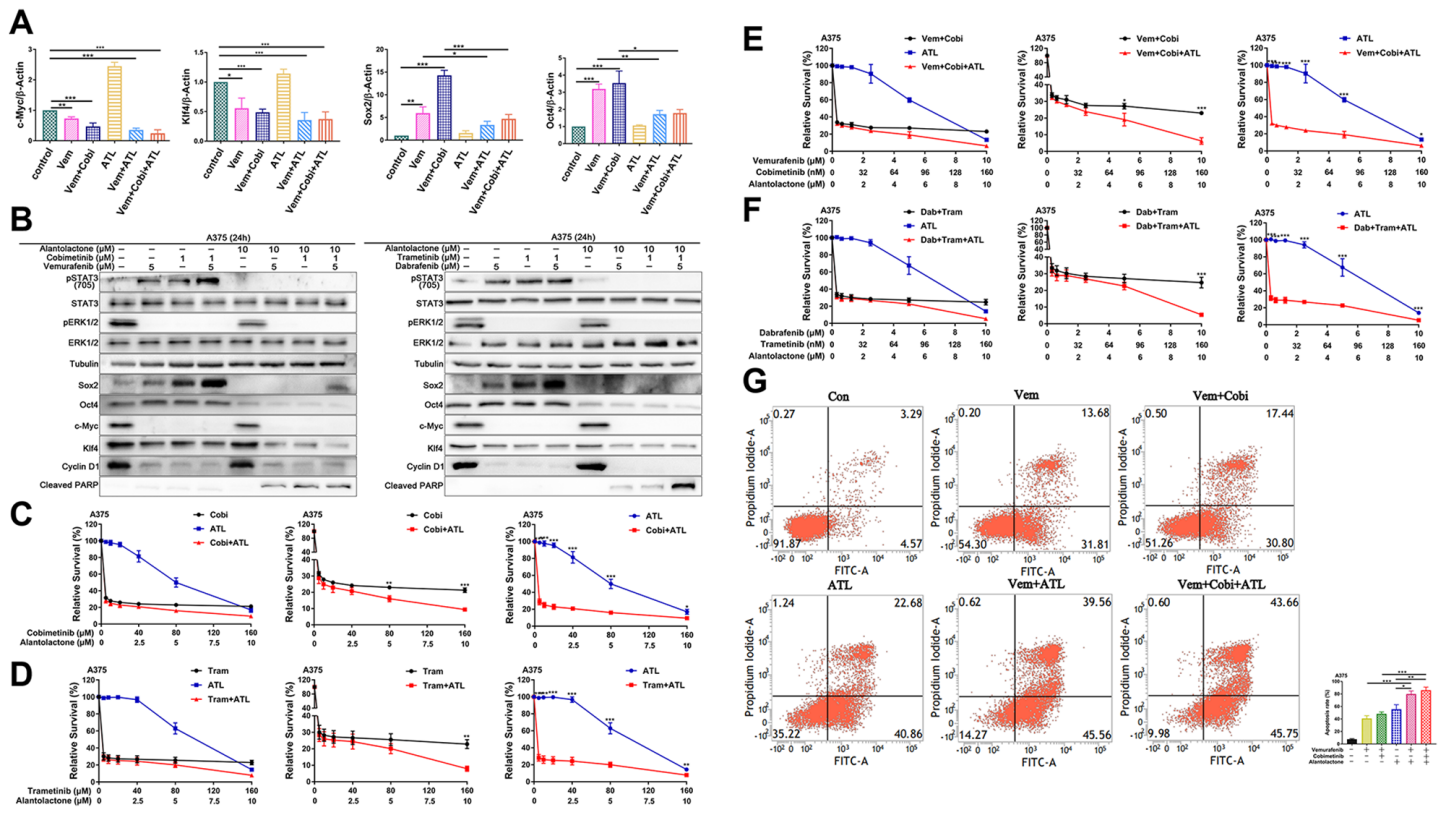
The combination of alantolactone and MAPKi simultaneously inhibited the STAT3 and BRAF/MEK/ERK pathways, regulating the expression level of downstream effectors in A375 cells. (**A**) A375 cells were treated with vemurafenib, cobimetinib and alantolactone alone or in combination for 6 h. The gene expression levels of c-Myc, Klf4, Sox2 and Oct4 were detected by real-time fluorescent quantitative PCR, and β-actin served as a loading control. (**B**) A375 cells were treated with vemurafenib, cobimetinib and alantolactone for 24 h. Phospho-STAT3 (705), STAT3, phospho-ERK1/2, ERK1/2, Sox2, Oct4, c-Myc, Klf4, cyclin D1 and cleaved PARP levels were analyzed by western blotting, and tubulin served as a loading control. (C and D) A375 cells were treated with different concentrations of cobimetinib and alantolactone (**C**) or trametinib and alantolactone (**D**) for 72 h. Cell viability was determined by CCK8 assays. The two images on the left in Figures C and D are split from the image on the right. (E and F) A375 cells were treated with different concentrations of vemurafenib, cobimetinib, and alantolactone (**E**) or dabrafenib, trametinib and alantolactone (**F**) for 72 h. Cell viability was determined by CCK8 assays. The two images on the left in Figures C and D are split from the image on the right. (**G**) A375 cells were treated with vemurafenib (5 µM), cobimetinib (1 µM), and alantolactone (6 µM) alone or in combination for 24 h. Flow cytometry analysis of cell death (Annexin V/PI labelling) in A375 cells. The histogram on the right shows the proportion of dead cells. Data, means ± SDs. *p < 0.05; **p < 0.01; ***p < 0.001; Student’s t test

Based on the above findings, we further confirmed the functions and the underlying mechanism of alantolactone in A375 cells. The mRNA and protein expression level of c-Myc, Klf4, Sox2 and Oct4 were compared analyzed. In cells of single MAPKi treatment, Oct4 and Sox2 were upregulated along with pSTAT3 (705) level increasing, while c-Myc and Klf4 were downregulated (Fig. 2A and B). Whereas pSTAT3 (705) level was sharply diminished in cells incubated with single alantolactone or alantolactone combined with MAPKi. In addition, Oct4 and Sox2 were decreased with the downregulation of pSTAT3 (705) in cells exposure to alantolactone and MAPKi compare with MAPKi alone (Fig. 2A and B). These results suggested alantolactone suppressed STAT3 activation, downregulating mRNA and protein expression of downstream Oct4 and Sox2 to anticancer.

To gather more evidences supporting the effectiveness of the combined treatment, we conducted cell proliferation experiments and cell apoptosis detection. The apoptosis marker Cleared PARP was increased in cells of alantolactone combined with MAPKi treatment compared with single drug (Fig. 2B). Comparison of the cell survival rate among various groups, it was significantly decreased in cells of alantolactone combined BRAFi + MEKi treatment(Fig. 2C-F). Flow cytometry assay results were consistent with the western blotting, indicating in cells of combined drugs exposure, apoptosis was significantly increased (Fig. 2G). These results suggested that alantolactone suppressed STAT3 activation, downregulating mRNA and protein expression of downstream Oct4 and Sox2 to anticancer.

### Alantolactone was highly selective cytotoxic effects on A375 cells, no obvious cytotoxicity on HK-2 cells

**Fig. 3:**
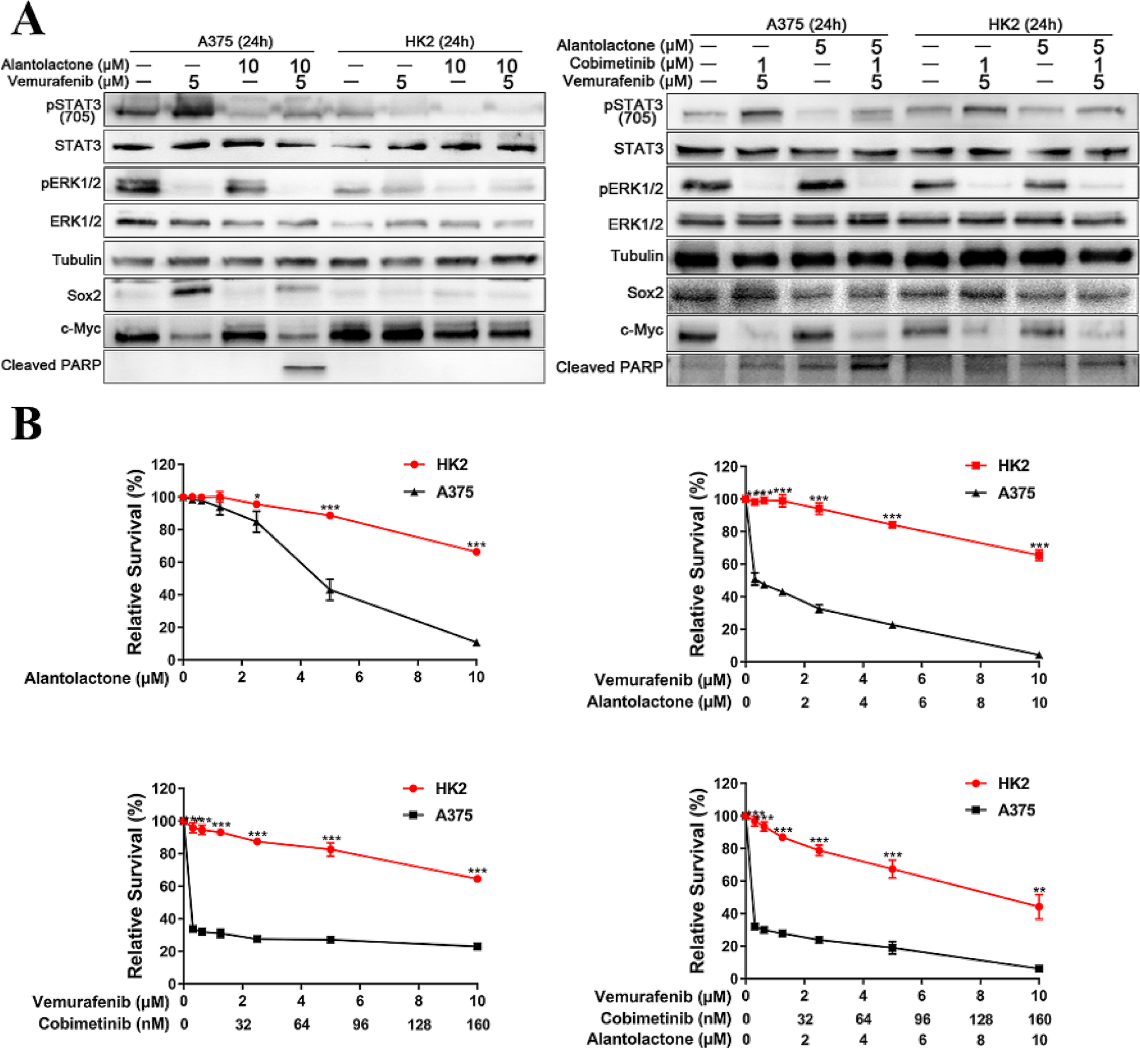
Combined treatment with alantolactone and MAPK pathway inhibitors showed highly selective cytotoxic effects on A375 cells, but had no obvious cytotoxicity on renal tubular epithelial cells. (**A**) HK2 and A375 cells were treated with vemurafenib and alantolactone or vemurafenib, cobimetinib and alantolactone for 24 h. Phospho-STAT3 (705), STAT3, phospho-ERK1/2, ERK1/2, Sox2, c-Myc and cleaved PARP levels were analyzed by western blotting, and tubulin served as a loading control. (**B**) HK2 and A375 cells were treated with different concentrations of alantolactone, vemurafenib and cobimetinib for 72 h. Cell viability was determined by CCK8 assays. Data are the mean ± SD. *p < 0.05; **p < 0.01; ***p < 0.001; Student’s t test

Given the importance of alantolactone combined kill effects with BRAFi + MEKi therapy on A375 cells, we next asked if alantolactone had cytotoxic effects on normal cells. Normal renal tubular epithelial HK-2 and A375 cells were treated by alantolactone, BRAFi and MEKi sigle or combined. We noticed that although both STAT3 and BRAF/MEK/ERK1/2 pathway were inhibited in A375 and HK-2 cells after 24 h exposure, but Cleared PARP were much higher in A375 than HK-2 cells (Fig. 3A). The survival rate decrease was significantly more in A375 cells than in HK-2 cells when treated with alantolactone combined with MAPKi (Fig. 3B). The results verified that alantolactone was highly selective enhancing the cytotoxic effects on A375 cells, no obvious side effects on HK-2 cells.

### Alantolactone had cooperating therapeutic effects with MAPKi by inhibiting STAT3, downregulating Oct4 and Sox2 in A375 xenografts of nude mice

**Fig. 4:**
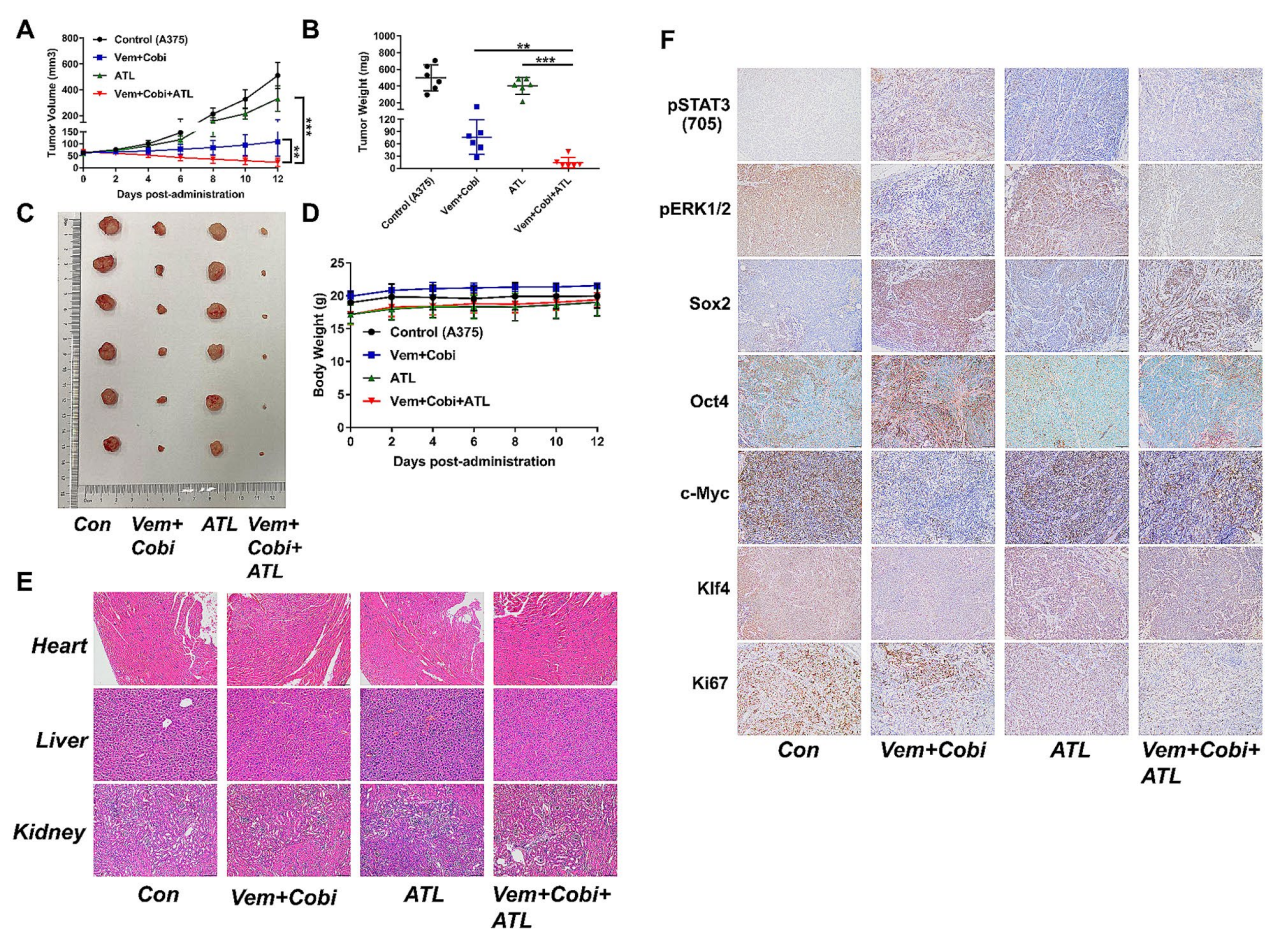
Alantolactone could synergistically enhance the cytotoxic effects with MAPKi in A375 xenografts of nude mice. (**A**) A375 xenografts were administrated by alantolactone (ATL; 20 mg/kg administered intraperitoneally once daily) and vemurafenib (Vem; 25 mg/kg administered intraperitoneally once daily) + cobimetinib (Cobi; 1 mg/kg administered intraperitoneally once daily) individually or in combination. During the treatment period, measure and record the tumor volume every other day. (**B**) After 12 days of treatment, tumor grafts were removed and weighed. (**C**) Photographs of xenograft A375 tumors treated with single or combination drugs. (**D**) During the drug treatment, the animals were weighed every other day. (**E**) Haematoxylin-eosin staining of heart, liver and kidney tissue sections (magnification: ×100). (**F**) Representative images of immunohistochemical staining of p-STAT3(705), p-ERK1/2, c-Myc, Klf4, Sox2, Oct4 and Ki67 in tumor tissues. Data are the mean ± SD. *p < 0.05; **p < 0.01; ***p < 0.001; Student’s t test

To confirm the antitumor effects of alantolactone in vivo, we established a xenograft model of A375 cells. When the tumors were approximately 75 mm [3], drug administrated by alantolactone and BRAFi + MEKi single or combined for 12 days. We tested the growth inhibitory effects of alantolactone in xenografts. Consistent with our previous findings in vitro studies, alantolactone combined BRAFi + MEKi caused significant tumor regression of A375 xenografts, compared with the groups of alantolactone or BRAFi + MEKi administration alone (Fig. 4A-C). The results confirmed alantolactone had cooperating therapeutic effects with BRAFi + MEKi on BRAF mutant melanoma in vivo. Within drug administration, we could not find any mice death and weight change before and after treatment (Fig. 4D). Damages of heart, liver and kidney of mice were observed under microscope by HE staining. We could not find swelling, steatosis, inflammatory infiltration and necrosis, and so on in these organs (Fig. 4E).

**Fig. 5:**
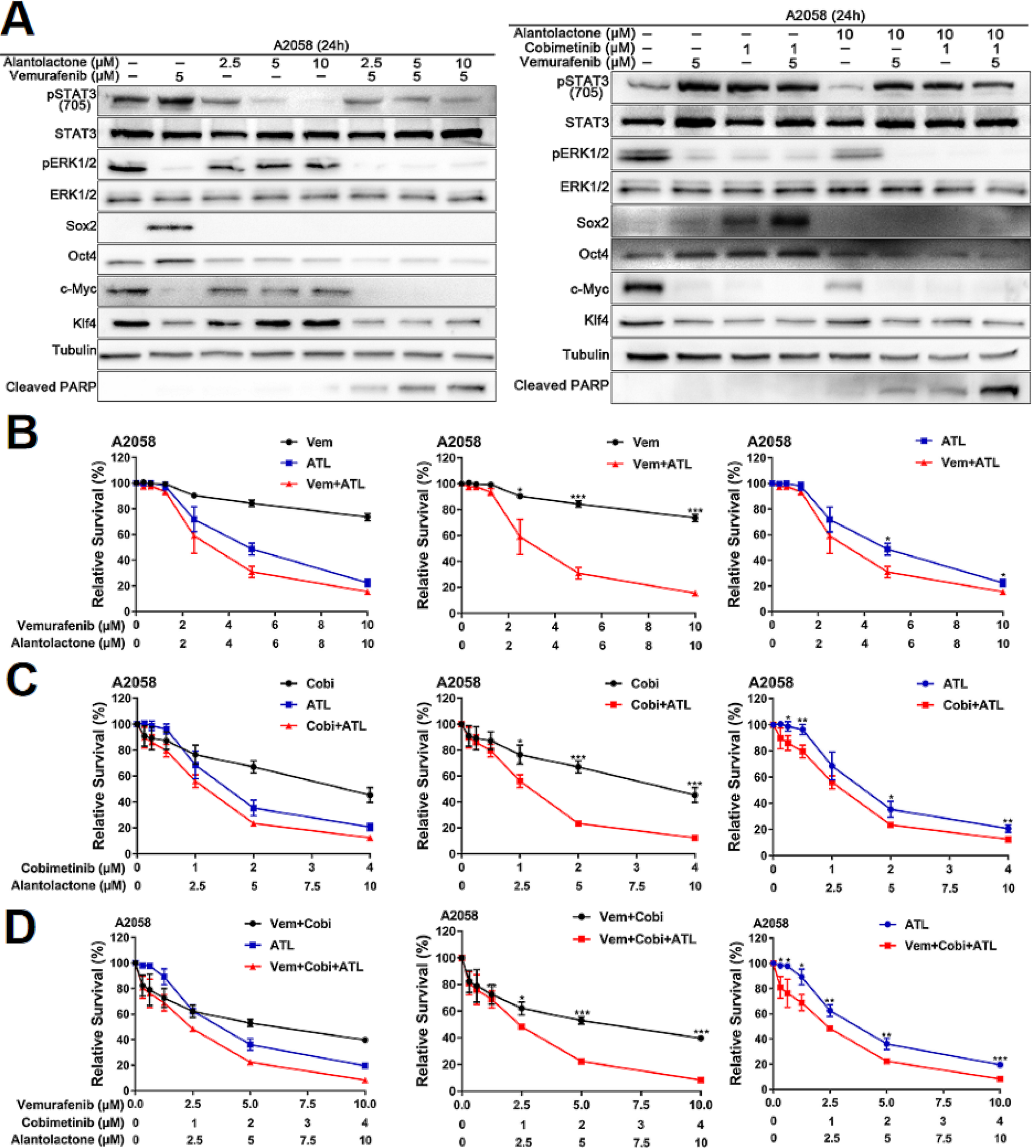
Alantolactone sensitized intrinsic-resistant A2058 cells to MAPKi targeted therapy by inhibiting STAT3 activation. (**A**) A2058 cells were treated with vemurafenib and alantolactone or vemurafenib, cobimetinib and alantolactone for 24 h. Phospho-STAT3 (705), STAT3, phospho-ERK1/2, ERK1/2, Sox2, Oct4, c-Myc, Klf4, cyclin D1 and cleaved PARP levels were analyzed by western blotting, and tubulin served as a loading control. (B-D) A2058 cells were treated with different concentrations of vemurafenib and alantolactone (**B**), cobimetinib and alantolactone (**C**), or vemurafenib, cobimetinib and alantolactone (**D**) for 72 h. Cell viability was determined by CCK8 assays. Data are the mean ± SD. *p < 0.05; **p < 0.01; ***p < 0.001; Student’s t test

To determine mechanisms of alantolactone in BRAF mutant melanoma in vivo, the proteins expression level of pSTAT3 (705), pERK1/2, c-Myc, Klf4, Oct4, Sox2 and Ki67 in tumors were detected by IHC testing. We found pSTAT3 (705), Oct4 and Sox2 were intensive in group MAPKi-treated, decreased in group alantolactone-treated, and all the pSTAT3 (705), pERK1/2, c-Myc, Klf4, Sox2, Oct4 and Ki67 were marked reduced in group treated with alantolactone combined MAPKi (Fig. 4F). These results confirmed that STAT3 activation was induced by MAPKi, however, alantolactone could inhibit STAT3 and its downstream effectors Oct4 and Sox2 activation. Therefore, alantolactone combined MAPKi could dual suppress STAT3 and BRAF/MEK/ERK1/2 pathway, consequently inhibiting the expression of downstream Sox2, Oct4, c-Myc and Klf4 proteins expression.

### Alantolactone sensitized intrinsic-resistant A2058 cells to MAPKi targeted therapy by inhibiting STAT3, downregulating Oct4 and Sox2 expression

Drug resistance is the biggest challenge in targeted therapy of melanoma. We want to know if the combination of alantolactone and MAPKi can be effective against drug-resistant melanoma. The Cancer Cell Line Encyclopedia (CCLE) and Catalogue of Somatic Mutations in Cancer (COSMIC) show that A2058 cells carry both BRAF and MEK mutations and are inherently drug-resistant melanoma cells. The results indicated that alantolactone could suppress STAT3 activation, downregulating Oct4 and Sox2 expression in A2058 cells (Fig. 5A). Alantolactone conjunction with BRAFi + MEKi administration, both STAT3 and BRAF/MEK/ERK1/2 pathways were inhibited, with significantly decreasing the downstream Klf4, Oct4, Sox2 and c-Myc (Fig. 5A). Cleaved PARP proteins was obvious descending, indicating alantolactone enhanced the killing effect of MAPKi on cancer cells (Fig. 5A). CCK8 results showed that the survival rate of A2058 cells were significantly reduced when alantolactone was combined with BRAFi + MEKi (Fig. 5B-D). These results clarified that alantolactone could inhibit STAT3 activation, downregulating Oct4 and Sox2 expression, raise response to BRAFi + MEKi targeted therapy in A2058 cells. These results raised the possibility of hypothesis that alantolactone combined MAPKi was a strategy to conquer drug resistance of melanoma to MAPKi targeted therapy.

### Alantolactone inhibited STAT3 signals, enhanced sensitiveness of acquired drug-resistant A375R cells to MAPKi

**Fig. 6:**
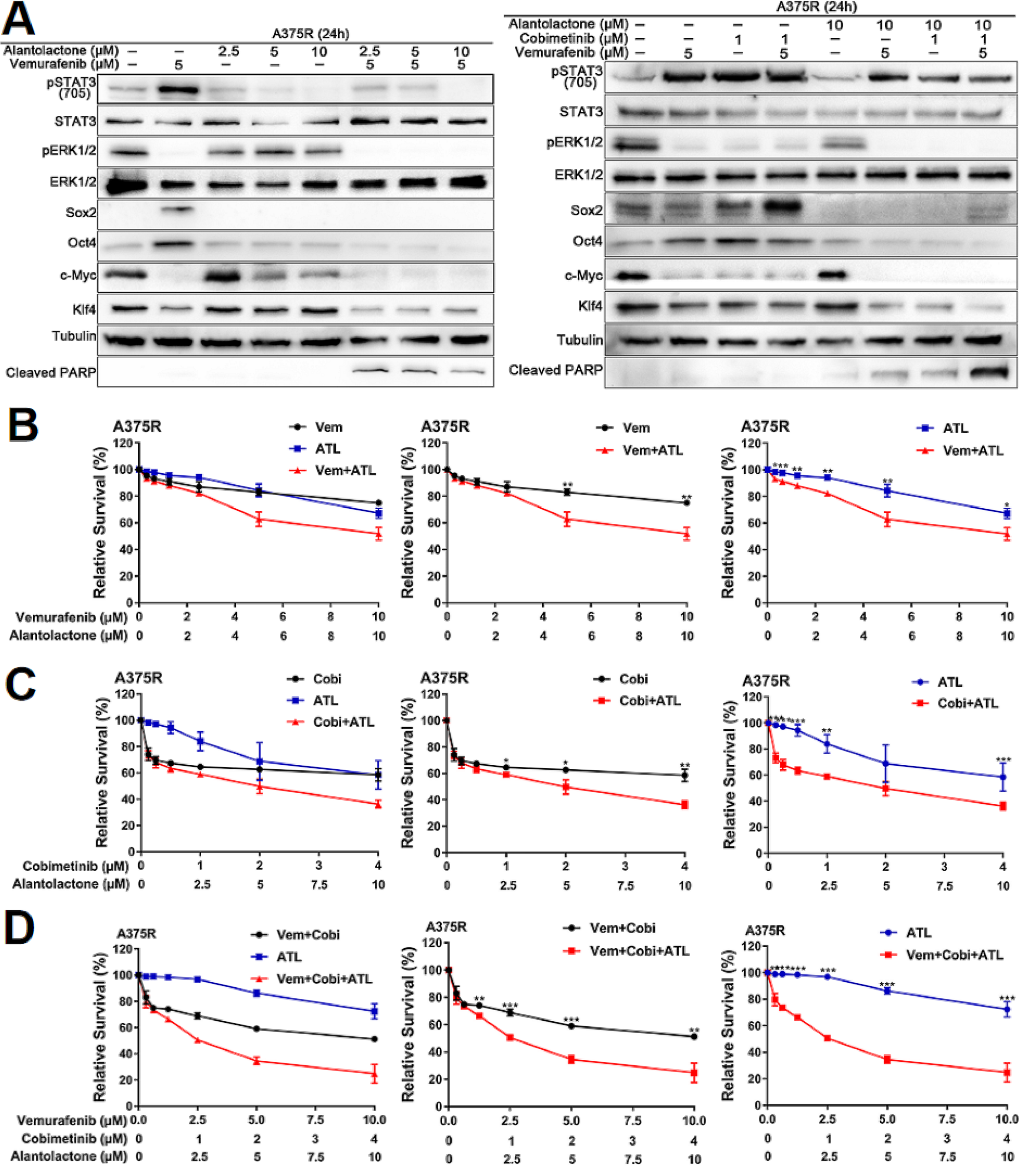
The combination of alantolactone and MAPKi could inhibit the proliferation of MAPKi-resistant A375R cells. (**A**) A375R cells were treated with vemurafenib and alantolactone or vemurafenib, cobimetinib and alantolactone for 24 h. Phospho-STAT3 (705), STAT3, phospho-ERK1/2, ERK1/2, Sox2, Oct4, c-Myc, Klf4, cyclin D1 and cleaved PARP levels were analyzed by western blotting, and tubulin served as a loading control. (B-D) A375R cells were treated with different concentrations of vemurafenib and alantolactone (**B**), cobimetinib and alantolactone (**C**), or vemurafenib, cobimetinib and alantolactone (**D**) for 72 h. Cell viability was determined by CCK8 assays. Data are the mean ± SD. *p < 0.05; **p < 0.01; ***p < 0.001; Student’s t test

Considering alantolactone had significantly enhanced intensiveness in intrinsic MAPKi-resistance melanoma, we should assess the effects and mechanisms by which alantolactone on acquired MAPKi-resistant of melanoma. A375R cell is a acquired drug-resistant cell model established by treating A375 cell with high dose BRAFi Vem. In order to further determine the effects of alantolactone on drug resistance to MAPKi, A375R cells were treated with alantolactone, BRAFi, MEKi and alantolactone alone or in combination. After 24 h treatment, the effects of alantolactone in A375R cells were totally consistent with those in A375 cells. MAPKi could induced STAT3 activation, and alantolactone inhibited STAT3. Alantolactone combined with BRAFi + MEKi simultaneously suppressed STAT3 and BRAF/MEK/ERK1/2 signals, downregulating the expression level of Oct4, Sox2, c-Myc, and Klf4, promoting cells apoptosis, inhibiting cells growth (Fig. 6A-D). It was suggested that alantolactone could enhance sensitiveness of A375R cells to MAPKi.

### Alantolactone enhanced sensitiveness of acquired drug-resistance to MAPKi in xenografts of A375R cells by inhibiting STAT3 signals

**Fig. 7:**
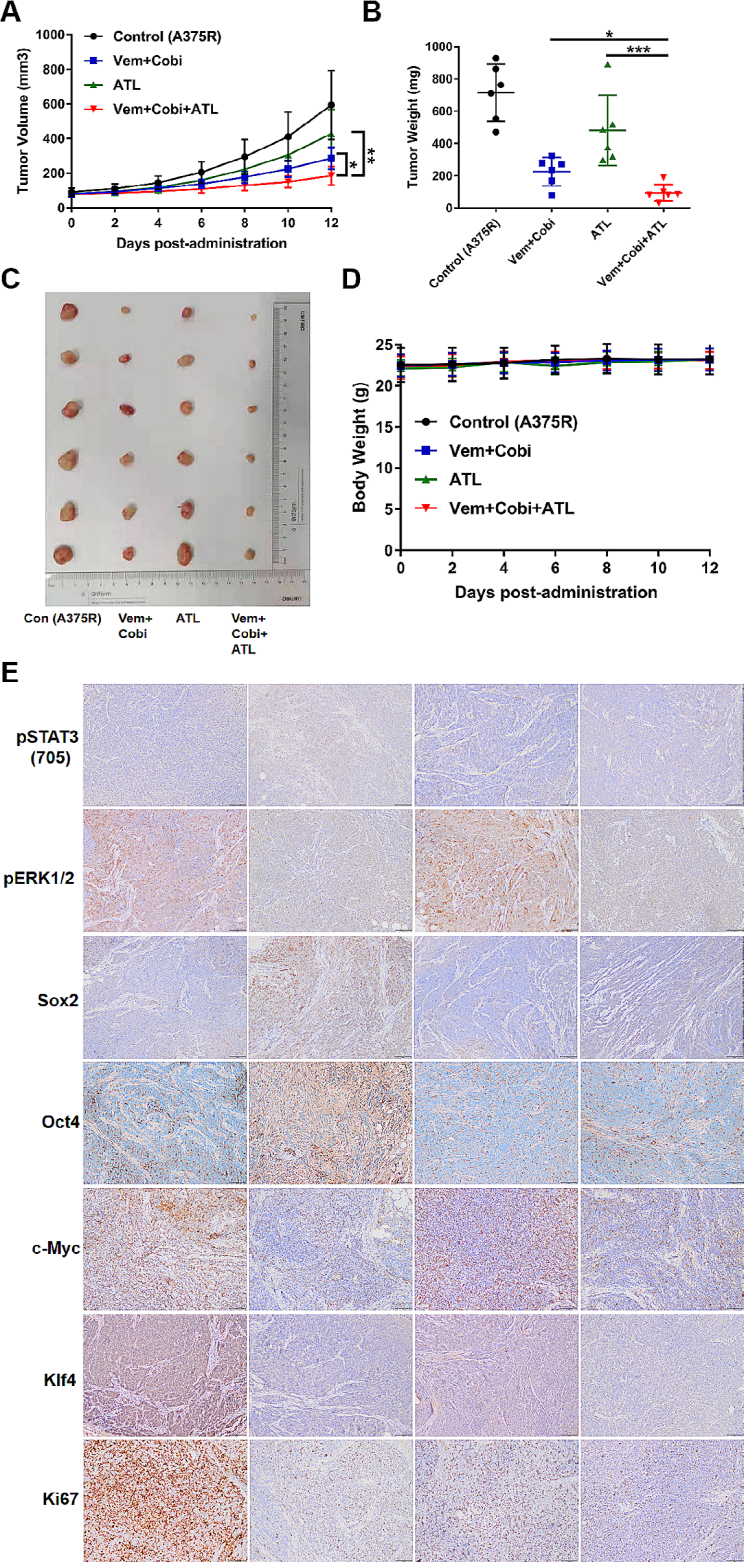
Alantolactone could synergistically enhance the cytotoxic effects with MAPKi in A375 xenografts of nude mice. (**A**) Tumor growth curves of A375R xenograft models treated with alantolactone (ATL; 20 mg/kg administered intraperitoneally once daily) and vemurafenib (Vem; 25 mg/kg administered intraperitoneally once daily) + cobimetinib (Cobi; 1 mg/kg administered intraperitoneally once daily) alone or in combination. (**B**) After 12 days of treatment, tumor grafts were removed and weighed. (**C**) Photographs of xenograft A375R tumors treated with single or combination drugs. (**D**) During the drug treatment, the animals were weighed every other day. (**E**) Representative images of immunohistochemical staining of p-STAT3(705), p-ERK1/2, c-Myc, Klf4, Sox2, Oct4 and Ki67 in tumor tissues. Data are the mean ± SD. *p < 0.05; **p < 0.01; ***p < 0.001; Student’s t test

To confirm the antitumor effects of alantolactone to acquired MAPKi-resistance in vivo, a xenograft model of A375R cell was established. Xenografts were administrated with alantolactone and BRAFi + MEKi single or combined for 12 days. Alantolactone combined BRAFi + MEKi significantly inhibited xenografts growth compared with the groups of alantolactone or BRAFi + MEKi administration alone (Fig. 7A-C). Any mice death and weight change before and after treatment could not been found (Fig. 7D). The results verified alantolactone enhanced sensitiveness of BRAF mutant melanoma to MAPKi in vivo.

The proteins expression level of pSTAT3 (705), pERK1/2, c-Myc, Klf4, Oct4, Sox2and Ki67 in tumors were detected by IHC testing. pSTAT3 (705) was marked intensive in group MAPKi treatment, decreased in group alantolactone-treated. After alantolactone combined with MAPKi treatment, STAT3 and its downstream protein Oct4 and Sox2, BRAF/MEK/ERK1/2 and its downstream protein c-Myc and Klf4, were prominently reduced (Fig. 7E). These results confirmed that STAT3 activation was a cause of MAPKi-resistance in BRAF mutant melanoma, however, alantolactone could inhibit STAT3 activation. Therefore, alantolactone combined MAPKi could dual suppress STAT3 and BRAF/MEK/ERK1/2 pathways, consequently inhibiting the expression of downstream Klf4, Oct4, Sox2 and c-Myc proteins, to enhance sensitivity of resistant melanoma to MAPKi.

## Discussion

Drug-resistance is a crucial obstacle of MAPKi treatment successful in advanced melanomas with BRAF mutant. Exploring the mechanisms and discovering approaches to overcome resistance is urgent. Numerous mechanisms of resistance have been predicted, detected and confirmed on pre and post-treatment tumor samples. Mutations of NRAS, HOXD8 or MEK, alterations of PI3K/AKT pathway, loss of PTEN, upregulation of FOXD3, MITF amplification and relief of MITF suppresion had been reported [26–31]. Earlier studies from others and our group have shown that STAT3 signaling activation may be a mechanism resisting to MAPKi [15, 17, 18, 32]. Our experiments here first verified previous findings again, which was if MAPKi could activate STAT3. Experiments performed on A375, vemurafenib-resistant A375 (A375R), intrinsic-resistant A2058 cells and their xenografts of nude mice. In vitro and vivo, the data confirmed that BRAFi, MEKi or BRAFi + MEKi administration alone could conspicuously elevate the protein level of pSTAT3 (705), activate STAT3 signaling, suppressing tumor apoptosis.

We further inquired into the molecules involved in STAT3 signals in MAPKi-resistance. Oct4 and Sox2 are transcription factors critical for maintaining stem cell pluripotency, which form a complex can act on downstream genes [33, 34]. Klf4 is a transcription factor acting as an oncogene or a tumor suppressor gene in diverse types of cancers [35]. c-Myc is an oncogene, which was upregulated in a variety of cancer types, participating in cell proliferation and differentiation [36]. Oct4, Sox2, c-Myc and Klf4 had been identified as cancer stem cell (CSCs) markers, playing a key role in the reprogramming of somatic differentiated cells into induced pluripotent stem cells (iPSCs) [37, 38]. We tested mRNA and protein expression of Oct4, Sox2, c-Myc and Klf4 in A375, A375R, A2058 cells and their xenografts of nude mice. mRNA and protein expression of Oct4 and Sox2 were concomitant with pSTAT3 (705). If cells or xenografts were treated with MAPKi alone, pSTAT3 (705), Oct4 and Sox2 were increased, suppressing apoptosis, and c-Myc and Klf4 were desended. Studies reported that overexpression of Oct4 and Sox2 were associated with drug-resistance, including chemotherapy, radiation therapy and target therapy in lung cancer and bladder cancer, malignant mesothelioma cells and osteosarcoma [39–42]. Ikushima et al. showed that Oct4 and Sox2 are critical in retaining tumorigenicity, and that knock-down of Oct4 increased the sensitivity of glioma cells to temozolomide [43]. Our data was consistent with previous studies, and we confirmed that MAPKi activates STAT3 signaling, upregulating the reprogramming factors Oct4 and Sox2 to resist MAPKi in melanomas.

Alantolactone was confirmed to possess STAT3 inhibition property and great potential for pancreatic cancer, breast cancer treatment [44–46]. In present study, we verified alantolactone could inhibited STAT3 activation, downregulating Oct4 and Sox2 expression induced by MAPKi feedback in A375, A375R and A2058 cells and their xenografts of nude mice. Alantolactone could enhance sensitiveness of melanoma to MAPKi. Dual targeting STAT3 and BRAF/MEK/ERK1/2 with alantolactone and MAPKi could enhance cytotoxic effects of cells and xenografts to MAPKi treatment.

In additional, we assessed the side effects of alantolactone. Data demonstrated that alantolactone was highly selective cytotoxic effects on A375 cells, no obvious cytotoxicity on normal renal tubular epithelial HK-2 cells. Experiments performed on xenografts of nude mice showed that damages of swelling, steatosis, inflammatory infiltration and necrosis could not been found in heart, liver and kidney.

In a conclusion, we first proposed STAT3 signaling activation by MAPKi, upregulated CSCs markers Oct4 and Sox2 as a mechanism of MAPKi-resistance in melanoma. Our results first suggested that STAT3-targeted therapy of alantolactone combined with MAPKi is a new therapeutic strategy to overcome resistance to MAPKi in the treatment of melanoma.

### Electronic supplementary material

Below is the link to the electronic supplementary material.

Supplementary Material 1

## Data Availability

All data are available in the main text or the supplemental information.
