# Supplementary material for: Alantolactone enhances the sensitivity of melanoma to MAPK pathway inhibitors by targeting inhibition of STAT3 activation and down-regulating stem cell markers
Source: Cancer Cell Int. 2024 May 31;24:191. doi: 10.1186/s12935-024-03371-9 (PMC11143683; doi:10.1186/s12935-024-03371-9)
Supplement: Supplementary file 1 — Supplementary Material 1 [file 12935_2024_3371_MOESM1_ESM.docx]

| **Gene** | **Forward Primer (5’-3’)** | **Reverse Primer (5’-3’)** |
| --- | --- | --- |
| β-actin | CTCCATCCTGGCCTCGCTGT | GCTGTCACCTTCACCGTTCC |
| SOX2 | AATGGGAGGGGTGCAAAAGAGG | GTGAGTGTGGATGGGATTGGTG |
| OCT4 | TTCGCAAGCCCTCATTTCAC | CCATCACCTCCACCACCTG |
| MYC | CGACGAGACCTTCATCAAAAAC | CTTCTCTGAGACGAGCTTGG |
| KLF4 | CAGCTTCACCTATCCGATCC | GAATGTACACCGGGTCCAATTC |

**Supplementary Table 1**: Primer sequences for RT-PCR experiments
